# Intervention time decides the status of autophagy, NLRP3 activity and apoptosis in macrophages induced by ox‐LDL

**DOI:** 10.1186/s12944-022-01714-x

**Published:** 2022-10-25

**Authors:** Liang Zheng, Hongbiao Xu, Fufu Zheng, Yuanhui Lai, Jie Li, Weiming Lv, Zuojun Hu, Wenjian Wang

**Affiliations:** 1 Laboratory of Department of Surgery, the First Affiliated Hospital of Sun Yat-Sen University, Guangzhou, 510080 Guangdong China; 2 Department of Thyroid and Breast Surgery, the First Affiliated Hospital of Sun Yat-Sen University, Guangzhou, 510080 Guangdong China; 3 Department of Urology, the First Affiliated Hospital of Sun Yat-Sen University, Guangzhou, 510080 Guangdong China; 4 Department of Thyroid and Breast Surgery, Guangzhou Women and Children’s Medical Center, Guangzhou Medical University, Guangzhou, 510623 Guangdong China; 5 Department of Vascular Surgery, the First Affiliated Hospital of Sun Yat-Sen University, Guangzhou, 510080 Guangdong China

**Keywords:** Atherosclerosis, Autophagy, Nucleotide-binding oligomerization domain-like receptor containing pyrin domain 3, Apoptosis; oxidized low-density lipoprotein, Macrophage

## Abstract

**Background:**

It has been determined through extensive studies that autophagy, the Nucleotide-binding oligomerization domain-like receptor containing pyrin domain 3 (NLRP3) inflammasome and apoptotic responses in macrophages jointly contribute to atherogenesis and its development in the presence of lipid abnormalities. Few studies have investigated in full-scale if the intervention time for lipids abnormality or NLRP3 activation have a significant effect on autophagy, NLRP3 or the apoptotic status in macrophages.

**Methods:**

Human THP-1 monocyte-derived macrophages were established by challenging THP-1 monocytes with 80 µg/ml oxidized low-density lipoprotein (ox-LDL) for specific durations. Foam cell formation was observed by Oil Red O (ORO) staining. Western blots were employed to determine protein expression. Transmission electron microscope (TEM) and immunofluorescence microscopy were applied to observe the autophagic status of cells. Cell apoptosis was evaluated by terminal deoxynucleotidyl transferase dUTP nick-end labeling (TUNEL).

**Results:**

The cells were treated with ox-LDL for 12 h and 36 h, which were considered to represent early and advanced stages of atherogenesis for this study. The results showed that inhibition of ox-LDL phagocytosis by cytochalasin D in the early stage improved autophagic status, reduced NLRP3 activation and the apoptotic response significantly. In contrast, cytochalasin D had little effect on blocking the detrimental effect of ox-LDL at the advanced stage. Moreover, the changes in autophagy, apoptosis and NLRP3 expression after treatment with small interfering (si) RNA targeting NLRP3 in the early and advanced stages of atherogenesis were consistent with the above data.

**Conclusions:**

Interventions against lipid disorders or inflammatory reactions in the early or advanced stages of atherogenesis may have different results depending on when they are applied during the process of atherosclerotic pathogenesis. These results may help improve therapeutic strategies for atherosclerosis prevention. Furthermore, a healthy lifestyle should still be recommended as the most important and inexpensive measure to prevent atherogenesis.

**Supplementary Information:**

The online version contains supplementary material available at 10.1186/s12944-022-01714-x.

## Introduction

It is well established that lipid dysregulation (mainly indicated by low-density lipoprotein cholesterol and its oxidative modification, ox-LDL), the ensuing inflammatory and apoptotic responses in macrophages jointly contribute to atherogenesis [1–5]. Dyslipidemia varies from childhood to adulthood, and both initiates and exacerbates atherosclerosis [6]. Lipids, with ox-LDL operating as the main factor, participate in the progression of atherosclerosis [2]. Longer lifetime exposure to lipid abnormalities is significantly associated with the severity of the atherosclerotic pathology [7].

Autophagy is a kind of catabolism in which cellular components such as ox-LDL are targeted and transferred to lysosomes for degradation [8]. Initially, ox-LDL is largely engulfed by macrophages [9, 10]. Thereafter, the lipid droplets are degraded through autophagy to avoid lipid accumulation within the cells and to maintain cellular homeostasis [11]. Deficiency of autophagy is closely linked to atherosclerosis [12]. Homeostasis is disrupted while the continuing lipid disorder and formation of excessive cellular lipid droplets are exacerbated, resulting in activation of lipid-based uncontrolled inflammatory pathways [13]. One of the major inflammatory signaling pathways leading to the development of atherogenesis involves the activation of the inflammasome [14]. The Nucleotide-binding oligomerization domain-like receptor containing pyrin domain 3 (NLRP3) inflammasome is the most extensively studied inflammasome [15]. Aberrant activation of the NLRP3 in macrophages causes apoptosis and boosts the development of atherogenesis [16–18]. Therefore, atherosclerosis is considered a chronic inflammatory disease [19, 20], in which macrophage foam cell formation and apoptosis predominate in its pathogenesis [5, 21].

Accordingly, drugs targeting lipid abnormality, inflammation and autophagy have been explored [22–24] for the alleviation of atherosclerosis. Currently, lipid-lowering drugs are widely prescribed in the clinic due to their great benefit in reducing rates of atherosclerotic cardiovascular disease morbidity and mortality [23, 25]. Nonetheless, atherosclerosis, after several decades of research on this pathology, is still the leading cause of mortality worldwide [26–29]. For this reason, seeking an overall understanding of the pathological process of atherosclerosis is necessary.

In the present study, autophagy, NLRP3 activity and apoptotic status were observed in macrophages at two different time points following exposure to ox-LDL and NLRP3 activation. Interventions against lipid disorders or inflammatory reactions in the early or advanced stages of atherogenesis were found to have different results depending on when they were applied during the process of atherosclerotic pathogenesis.

## Methods

### Materials

Ox-LDL was purchased from Yi-yuan Biotechnology (YB-002; Guangdong, China). Cytochalasin D was purchased from MCE (HY-N6682; Monmouth Junction, NJ, USA). Specific monoclonal antibodies against LC3II/I (#4108), SQSTM1/P62 (#8025), mTOR (#2983), phosphor-mTOR (Ser2448) (#5536), Beclin1 (#3495), ATG7 (#8558), Bcl2 (#4223), Bax (#5023), NLRP3 (#15,101) and GAPDH (#2118), as well as anti-rabbit IgG HRP-linked antibody (#7074) were bought from Cell Signaling Technology (Danvers, MA, USA). Lamp 1 (ab24170) was bought from Abcam (Cambridge, UK).

#### Cell culture and treatment

Human THP-1 monocytes were purchased from the American Type Culture Collection (Manassas, VA, USA). The cells were cultured in RPMI 1640 medium (Gibco, China) supplemented with 10% fetal bovine serum (Gibco, Australia) and 1% penicillin/streptomycin (MRC, Cincinnati, OH, USA), and were kept at 37℃ in a humidified incubator with 5% CO_2_. THP-1 cells in the log phase were seeded in both 6-well and 24-well plates at a density of 1.0 × 10^5^ cells per well with 100 ng/mL phorbol-12-myristate-13-acetate (PMA; Sigma-Aldrich, St. Louis, MO, USA) for 48 h for differentiation into macrophages. To establish a cell model of atherogenesis, the macrophages were treated with ox‐LDL at different concentrations and durations.

#### Cell transfection

The differentiated THP-1 cells were allowed to get 50%–70% confluence at the time of transfection in 6-well plates. mRFP-GFP-LC3 adenoviral vectors were designed and synthesized by Hanbio (Shanghai, China), and transfected into cells for 48 h according to the manufacturer’s protocol. In other experiments, the cells were transfected with a mixture containing 5 μl of Lipofectamine RNAiMAX (Invitrogen, Carlsbad, CA, USA) and 5 μl of small interfering RNA (siRNA; RiboBio, Guangzhou, China) targeting NLRP3 in 500 μl of Opti-MEM (Gibco, China). After transfection, the cells were cultured with RPMI-1640 for another 24 h, and then the medium was changed to RPMI-1640 with or without the ox-LDL. The sense sequence of siRNA targeting NLRP3 (si-NLRP3) was 5'-GAAATGGATTGAAGTGAAA-3'.

#### Transmission electron microscopy (TEM)

To detect autophagosomes, cells were managed as described above. The detailed procedures were referred to the study [30].

### Western blot analysis

Protein samples were made from the lysates of cultured cells, and its concentration was detected by Bradford assay. 15–30 μg of total protein from each sample was loaded onto 8–12% polyacrylamide gels. After separation via SDS-PAGE, the samples were transferred to PVDF membranes, and the membranes were blocked with TBS containing 0.05% Tween-20 (TBST) and 5% nonfat milk powder for 2 h. Afterwards the membranes were treated with the specified primary antibodies overnight at 4℃. The membranes were then treated with corresponding secondary antibodies for 1 h at room temperature. The protein levels were observed through a chemiluminescent technique.

### Immunofluorescence assay

The cells were put on 13-mm diameter slides and fixed using cold paraformaldehyde (4%) at 4 °C for 20 min. Subsequently, the cells were incubated with a rabbit NLRP3 antibody (NBP2-12,446; Novus Biologicals, Centennial, CO, USA) at 4 °C overnight. Then the cells were treated with an Alexa Fluor® 488 donkey anti-rabbit IgG (H + L) secondary antibody (Invitrogen, USA) at room temperature for 1 h. Finally, the slides were observed by a Nikon microscope (Tokyo, Japan).

### Apoptosis assay

For apoptosis assays, cells were harvested and stained using a kit for terminal deoxynucleotidyl transferase dUTP nick end labeling (TUNEL; 0,420,200,721, Beyotime, China) according to the manufacturer’s instructions.

### Oil Red O (ORO) staining

After various interventions, macrophages were washed with PBS three times, and fixed with 4% paraformaldehyde for 10 min, then washed with PBS twice, followed by staining with ORO solution 30 min. The cells were washed with PBS three times again. ORO staining was observed under a light microscope and measured by ImageJ software (National Institutes of Health, Bethesda, MD, USA).

### Statistical analysis

All data were expressed as means ± standard error of the mean (SEM). All experiments were performed three times independently. Student’s *t*-test was employed for different analyses between groups. *P* value < 0.05 was considered statistically significant.

## Results

### The development of autophagy, the NLRP3 inflammasome and apoptosis in macrophages changes with different concentrations and durations of ox‐LDL incubation

**Fig. 1:**
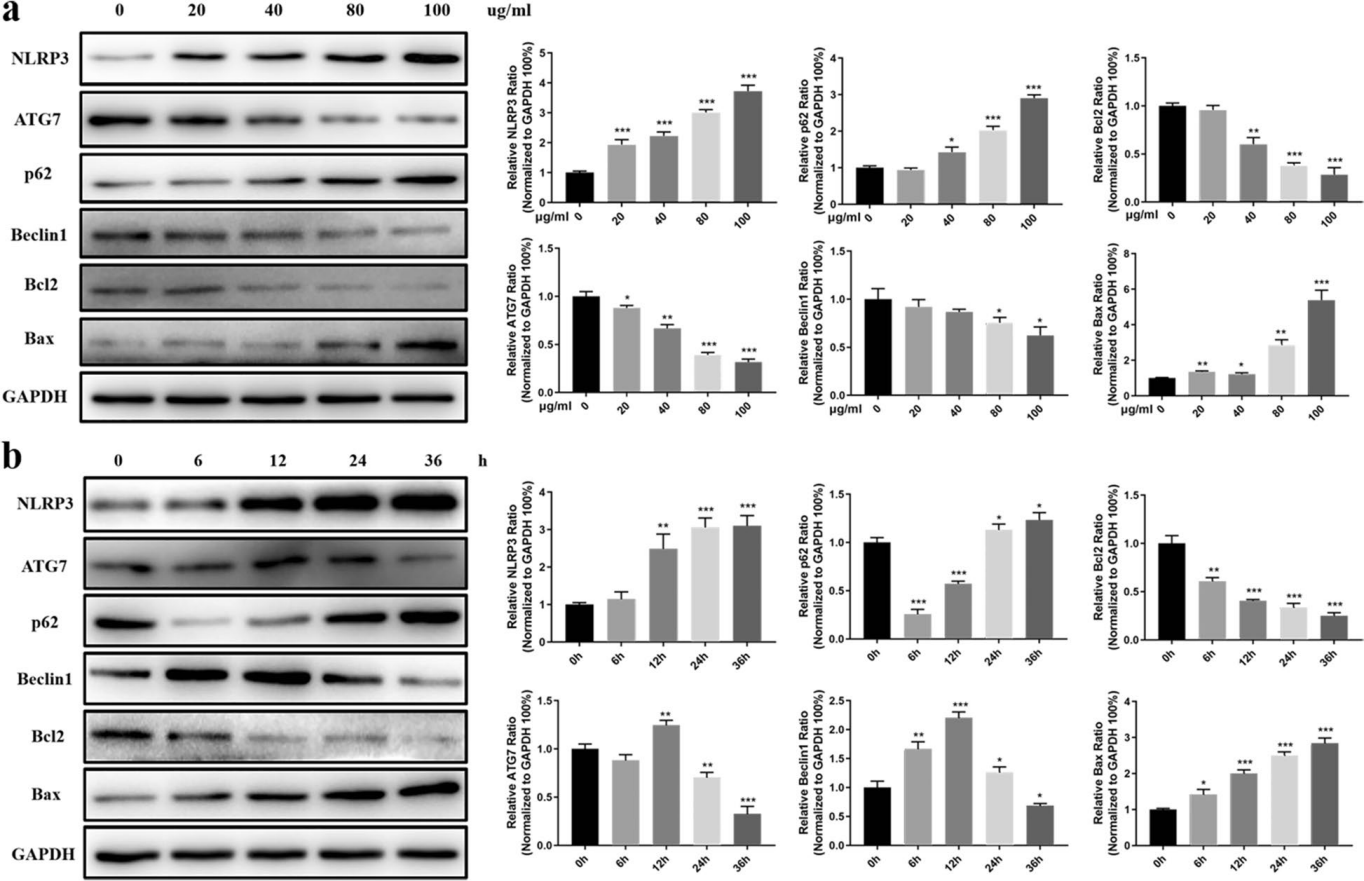
Variations in autophagy, NLRP3 levels and apoptosis in macrophages after exposure to different concentrations and durations of ox‐LDL incubation. **a** Macrophages were incubated with different doses of ox-LDL (0, 20, 40, 80 and 100 µg/ml) for 24 h. Western blot assays were applied for analyses of autophagy-related proteins, NLRP3 and apoptosis-related proteins expression. **b** Macrophages stimulated with 80 µg/ml ox-LDL for different time (0, 6, 12, 24 and 36 h). The changes in autophagy-related proteins, NLRP3 and apoptosis-related proteins were analyzed via western blot. *P < 0.05; **P < 0.01; ***P < 0.001

To choose a feasible ox‐LDL concentration and treatment time for the study, the development of autophagy as well as the NLRP3 inflammasome and apoptotic states in macrophages were investigated after exposure to different concentrations of ox‐LDL for different durations. Firstly, the macrophages were incubated with different doses of ox-LDL (0, 20, 40, 80 and 100 µg/ml) for 24 h. Western blot assays showed that expression levels of autophagy-related proteins (ATG7 and Beclin1) declined to their lowest level at an ox-LDL concentration of 80 µg/ml, indicating loss of the autophagic response, while NLRP3 activity increased in a dose-dependent manner. In addition, the apoptotic protein Bax was significantly increased in macrophages at ox-LDL concentration of 80 µg/ml (Fig. 1a). ORO staining revealed a concentration-dependent increase in the formation of foam cells (Fig. S1a). These results suggested that 80 µg/ml ox-LDL was sufficient to disrupt cellular autophagic capacity in 24 h. Therefore, because it was the commonly used dose of ox-LDL in the literature [31–33], 80 µg/ml ox-LDL was chosen to conduct the study.

Macrophages were then treated with 80 µg/ml ox-LDL for different durations (0, 6, 12, 24 and 36 h). Notably, the expression levels of ATG7 and Beclin1 increased significantly after 12 h but then declined after that, and were significantly decreased at 36 h. p62 began to increase at 12 h in a time-dependent manner. Simultaneously, the anti-apoptotic protein Bcl2 decreased most prominently at 12 h, while Bax was enhanced in a time-dependent manner after 12-h exposure. NLRP3 activity was increased after 12-h exposure in a time-dependent manner (Fig. 1b), as were the number of foam cells detected by ORO staining (Fig. S1b). The results indicated that autophagy could protect against lipid detrimental effects for only a limited time. Otherwise, the deterioration caused by inflammatory or apoptotic pathology occurred when prolonged lipid abnormality exceeded the capacity of autophagic protection. These results are in line with previous studies [16, 21]. Thus, 80 µg/ml ox‐LDL was adopted to perform the experiments, and 12 h and 36 h exposures to ox‐LDL were considered to represent the early and advanced stages, respectively, of lipid toxicity in this study.

### Inhibition of ox-LDL phagocytosis in the early and advanced stages of atherogenesis alters autophagic status in macrophages

**Fig. 2:**
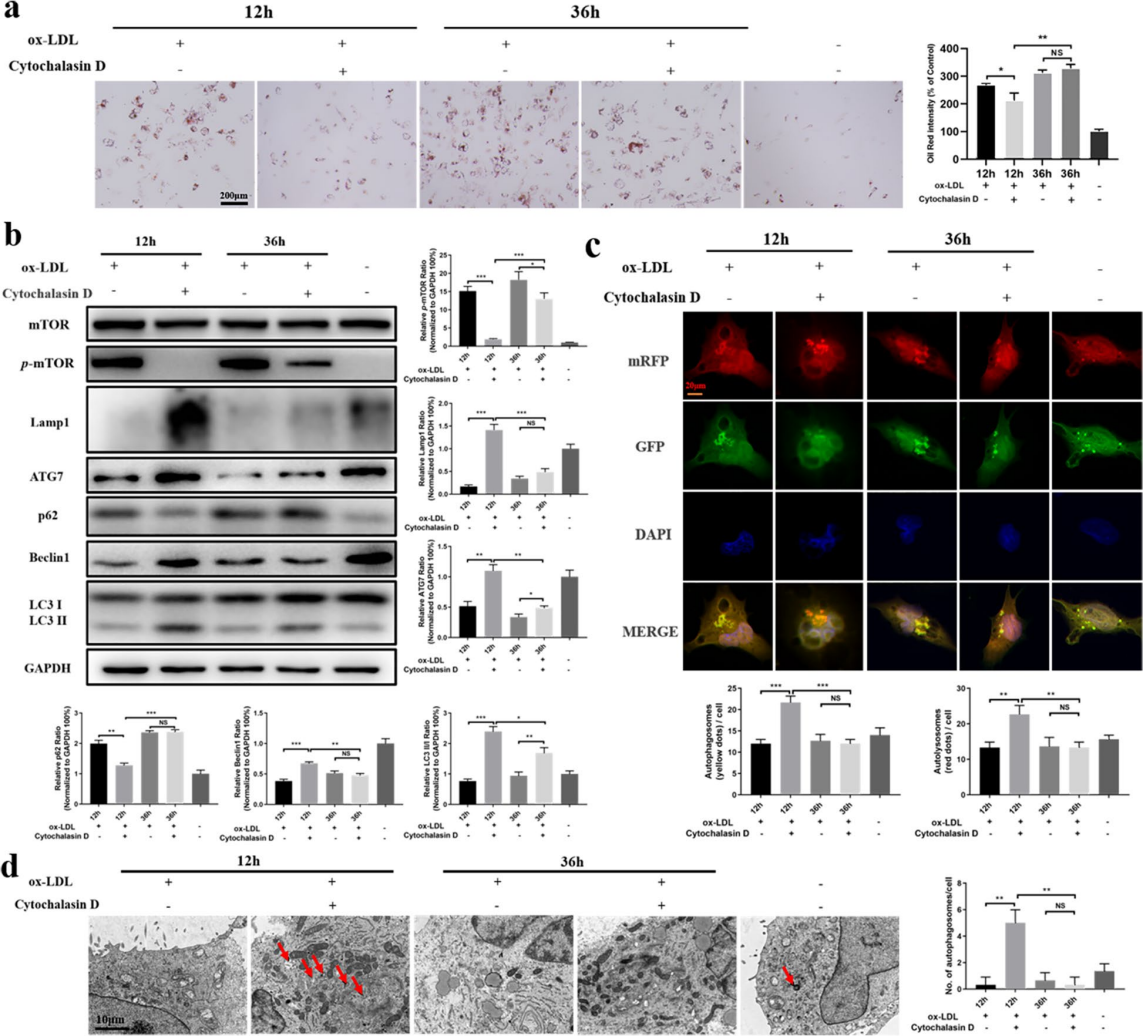
Inhibition of ox-LDL phagocytosis in the early or advanced stage of atherogenesis is associated with a different autophagic status in macrophages. **a** Intracellular lipid content was measured by ORO staining after the cells were treated with cytochalasin D (3 μM) at 12 h and 36 h. Scale bar = 200 μm. **b** Western blot analyses of autophagy-related proteins after blockage of ox-LDL phagocytosis at 12 h or 36 h. **c** Macrophages were transfected with an adenovirus expressing mRFP-GFP-LC3 to measure autophagic flux intensity quantitatively. Scale bar = 20 μm. **d** Autophagosomes (indicated by the red arrows) were observed by TEM. Scale bar = 10 μm. *P < 0.05; **P < 0.01; ***P < 0.001

The first issue that needed to be resolved by this study was whether autophagic status differed in macrophages in the early or advanced stage of lipid abnormality. To address this question, cytochalasin D (3 μM), an inhibitor of phagocytosis, was used to inhibit the effect of ox-LDL on macrophages. ORO staining assays showed that lipid deposition in cells was significantly reduced when phagocytosis of ox-LDL was blocked at 12 h. Although there were no differences in lipid deposition between groups with or without cytochalasin D at 36 h, the overall intensity of lipid deposition was much stronger at 36 h for both groups compared to 12 h (Fig. 2a). This result indicated that management of lipid abnormality at an early stage could prevent macrophage foam cell formation.

Subsequently, the changes in autophagy were examined in macrophages. Compared with the control groups, inhibition of ox-LDL phagocytosis at 12 h led to a significant increase in ATG7, Beclin1 and LC3 II/I expression, but a significant decrease in p62 or phosphorylated mTOR (*p*-mTOR) expression. Interestingly, although ATG7 and LC3 II/I expression were also significantly enhanced when phagocytosis of ox-LDL was blocked at 36 h, their expression was much less than in cells exposed at 12 h. In addition, there were no significant changes in p62 or Beclin1 expression when ox-LDL phagocytosis was blocked at 36 h, but the level of Beclin1 was reduced and the level of p62 increased dramatically compared to cells exposed at the 12 h time point (Fig. 2b). Furthermore, immunofluorescence microscopy was employed to examine autophagic variation in cells transfected with mRFP-GFP-LC3. Abundant formation of red puncta indicating the presence of autolysosomes was observed when ox-LDL phagocytosis was arrested in the early stage, but few red puncta were observed after arrest in the advanced stage (Fig. 2c). This was furtherly supported by the change of Lamp 1 (Fig. 2b). TEM was then used to observe the autophagosomes, and the results were similar to the immunofluorescence assays (Fig. 2d). These data suggested that the elimination of the lipid detrimental effect on macrophages in the early stage of atherogenesis could maintain the cells' autophagic capacity to prevent foam cell formation. However, once lipid abnormality exceeds a critical time period, autophagy fails to maintain cellular homeostasis and prevent atheropathogenesis, likely because drugs applied to counter the lipid disorder during this period make it hard to recover cellular autophagic capacity [24].

### NLRP3 activity changes following inhibition of ox-LDL phagocytosis in the early and advanced stages of atherogenesis

**Fig. 3:**
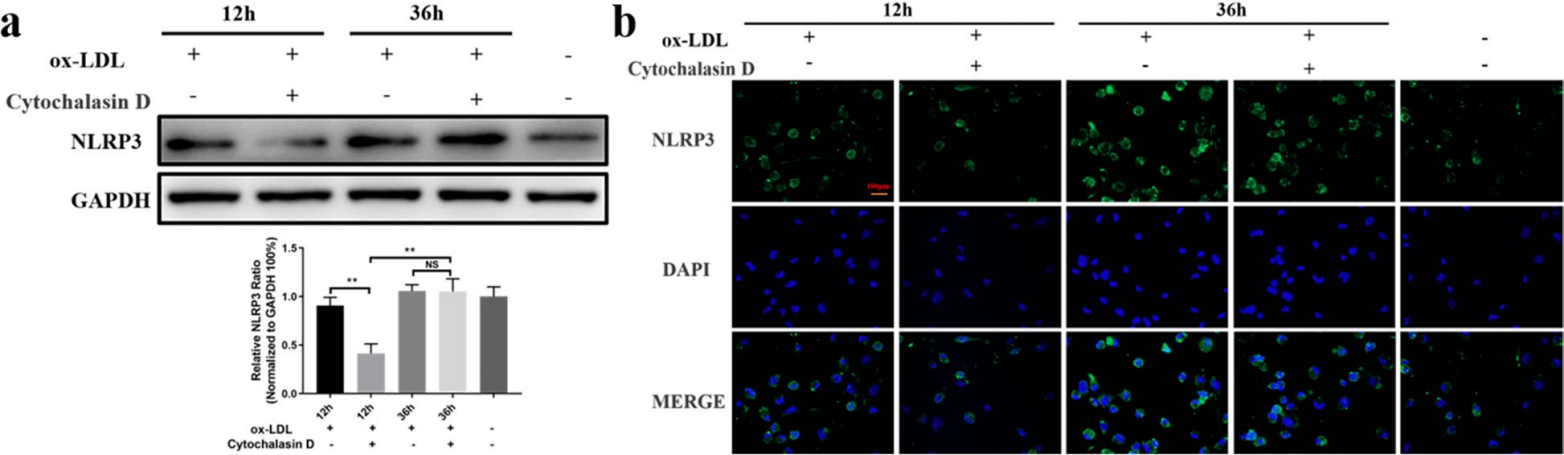
NLRP3 activity changes following inhibition of ox-LDL phagocytosis in the early or advanced stage of atherogenesis. **a** Western blot analyses of NLRP3 after inhibition of ox-LDL phagocytosis at 12 h and 36 h. **b** Immunofluorescence was used to assess the expression of NLRP3 (green fluorescence). DAPI-stained nuclei (blue) are also shown. Scale bar = 100 μm. **P < 0.01

It is well known that ox-LDL is a critical factor that contributes to the initiation and progression of atherosclerosis [2]. Ox-LDL can activate the NLRP3 signaling pathway in furtherance of atherogenesis [34]. Until now, few studies have examined the variation in NLRP3 activity caused by varying the intervention time for lipid disorders. The results from the present study showed that NLRP3 expression was significantly reduced when ox-LDL phagocytosis was blocked in the early stage. In contrast, NLRP3 expression showed little change compared with control cells when ox-LDL phagocytosis was suppressed in the advanced stage, although the NLRP3 level was much higher compared to the early stage (Fig. 3a). These findings were further supported by the results of immunofluorescence assays (Fig. 3b). The data indicated that removal of the ox-LDL detrimental effect at the early stage could efficiently attenuate inflammatory reactions; however, the detrimental effect of ox-LDL may be irreversible if its duration exceeds a critical time even if drugs for lipid disorders are applied at this period because of the uncontrolled flooded inflammation in the cells.

### Blockage of ox-LDL phagocytosis in the early and advanced stage of atherogenesis significantly alters apoptotic status in macrophages

**Fig. 4:**
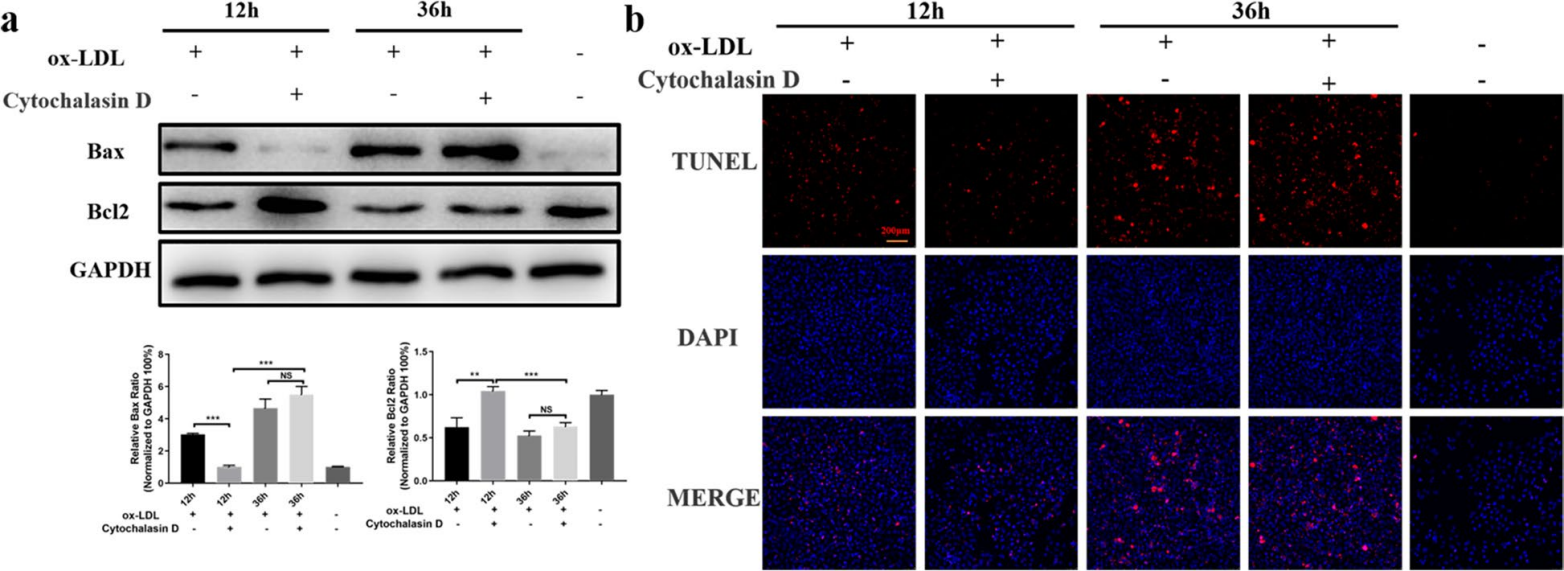
Inhibition of ox-LDL phagocytosis in the early or advanced stage of atherogenesis significantly alters apoptotic status in macrophages. **a** Western blot analyses of apoptosis-related proteins after blockage of ox-LDL phagocytosis in the early or advanced stages. **b** TUNEL assays show the number of apoptotic cells in different groups. Scale bar = 200 μm. **P < 0.01; ***P < 0.001

Ox-LDL-induced macrophage foam cell formation and apoptosis are critical mediators in the atherogenesis [21]. Therefore, clarifying the apoptotic status in macrophages when ox-LDL infliction is suppressed in the early or advanced stages is important for choosing therapeutic strategies for arteriosclerosis. As expected, western blot assays demonstrated that inhibition of ox-LDL phagocytosis in the early stage decreased Bax expression and elevated Bcl-2 expression significantly. Although inhibition of ox-LDL phagocytosis in the advanced stage did not affect Bax or Bcl-2 expression significantly compared with control cells, the levels of Bax and Bcl-2 were much higher and lower, respectively, than at of the early stage (Fig. 4a). Additionally, the results of TUNEL assays were in accordance with western blot assays (Fig. 4b). These results suggested that correction for lipid disorders in the early stage might prevent macrophage apoptosis. Otherwise, apoptosis may be nonreversible even if interventions against lipids abnormalities are applied in the advanced stage.

### Interference with NLRP3 expression in the early or advanced stage results in altered autophagic capacity in macrophages induced by ox‐LDL

**Fig. 5:**
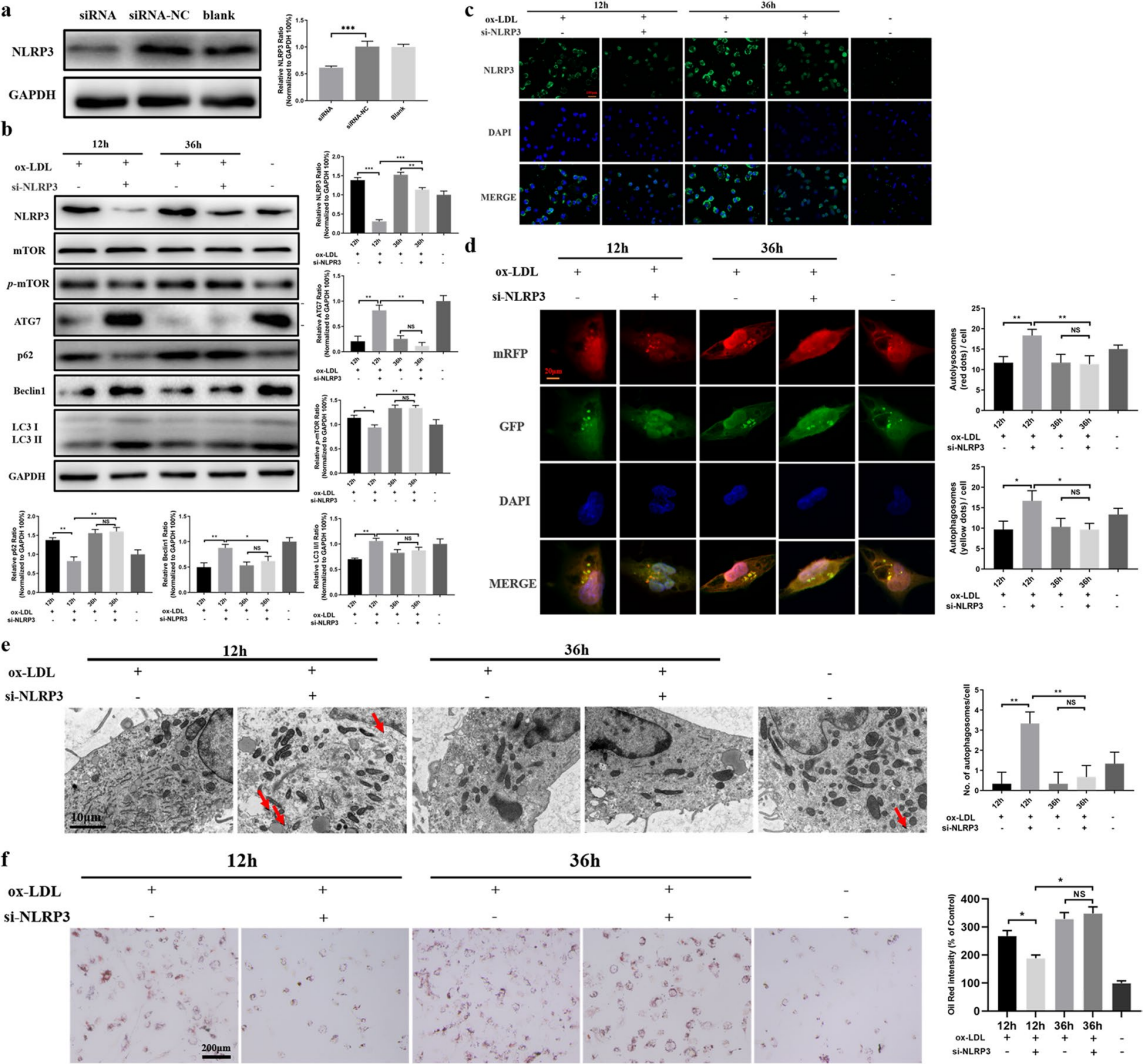
Application of si-NLRP3 in the early or advanced stage of atherogenesis alters autophagic capacity in macrophages induced by ox‐LDL. **a** Western blot was utilized to analyze NLRP3 expression after exposure to si-NLRP3. **b** Western blot analyses of NLRP3 and autophagy-related proteins after si-NLRP3 exposure in the early and advanced stage. **c** Immunofluorescence was used to assess the expression of NLRP3 (green fluorescence). DAPI-stained nuclei (blue) are also shown. Scale bar = 100 μm. **d** Macrophages were transfected with an adenovirus expressing mRFP-GFP-LC3 to measure autophagic flux intensity. Scale bar = 20 μm. **e** Autophagosomes (indicated by the red arrows) were detected by TEM. Scale bar = 10 μm. **f** Intracellular lipid droplets were observed using ORO staining after si-NLRP3 was administered in the early and advanced stage. Scale bar = 200 μm. *P < 0.05; **P < 0.01; ***P < 0.001

Considering that inflammasomes can regulate the autophagic status, and allowing for two-way mutual regulation of atherosclerotic inflammation [35], it is possible that interfering with NLRP3 expression in the early or advanced stage of atherogenesis may alter the autophagic status in macrophages incubated with ox-LDL. To test this hypothesis, siRNA was utilized to interfere with NLRP3 expression in macrophages (Fig. 5a). The experiments showed that application of si-NLRP3 in the early stage reduced NLRP3 expression significantly following ox-LDL stimulation. However, while NLRP3 expression was suppressed compared with control cells when si-NLRP3 was used at the advanced stage, NLRP3 expression was much higher than in the early stage (Fig. 5b-c). Moreover, the results revealed that the application of si-NLRP3 in the early stage increased expression levels of ATG7, Beclin1 and LC3 II/I, and decreased expression levels of *p*-mTOR and p62 significantly in the presence of ox-LDL incubation. Although the expression levels of the above autophagy-related proteins did not change significantly compared with the controls when si-NLRP3 was used in the advanced stage, the levels of ATG7, Beclin1 and LC3 II/I were downregulated, and the levels of *p*-mTOR and p62 were upregulated compared to the early stage (Fig. 5b). Immunofluorescence microscopy and TEM both displayed consistent changes in the number of autophagosomes (Fig. 5d-e). In addition, ORO staining showed that lipid droplets in cells were significantly reduced when si-NLRP3 was used at the early stage, but the number of droplets did not change appreciably when si-NLRP3 was employed in the advanced stage (Fig. 5f).

The above results suggested that early interference with inflammasome activation can not only inhibit development of inflammation but also help to efficiently recover autophagy status. Otherwise, excessive activation of inflammasomes over time can aggravate autophagy dysfunction, in which case autophagy would be inefficient at preventing atherogenesis at the advance stage [34]. These data are consistent with the literature [14, 36].

### Application of si-NLRP3 in the early and advanced stages of atherogenesis results in different apoptotic states in macrophages induced by ox‐LDL

**Fig. 6:**
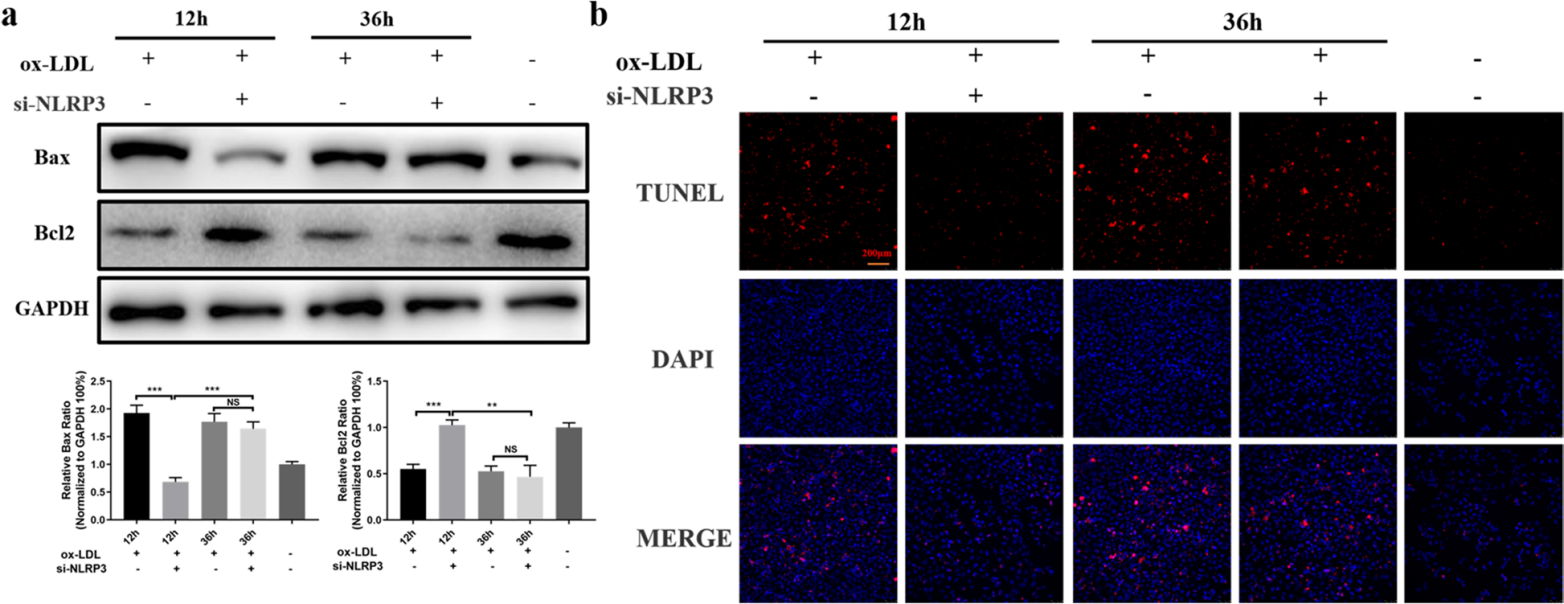
Knockdown of NLRP3 in the early and advanced stage of atherogenesis results in different apoptotic states in macrophages induced by ox‐LDL. **a** Western blot analyses of apoptosis-related proteins after si-NLRP3 was used in the early or advanced stage. **b** TUNEL assays were utilized to detect the number of apoptotic cells. Scale bar = 200 μm. **P < 0.01; ***P < 0.001

It has been confirmed that NLRP3 inflammasome activation can cause apoptosis of macrophages [16]. Therefore, it was hypothesized that the time of intervention against the NLPR3 reaction would affect the apoptotic status in macrophages. The application of si-NLRP3 in macrophages in the early stage reduced Bax expression and enhanced Bcl2 expression significantly. Although application of si-NLRP3 in the advanced stage did not significantly change the amount of Bax or Bcl2 compared with the controls, the level of Bax was nonetheless increased, while the level of Bcl2 was obviously decreased compared to cells in the early stage (Fig. 6a). TUNEL assays showed similar results (Fig. 6b). These results suggested that NLRP3 activity was closely related to apoptotic status in macrophages induced by ox-LDL [16]. Inhibition of NLRP3 activation at the early stage may efficiently block stimulation of apoptosis. However, interference with the NLRP3 reaction in the advanced stage appears to have little impact on apoptotic status.

## Discussion

Multiple lines of evidence indicate that dyslipidemia, macrophage autophagy and inflammatory reactions are closely associated with the initiation and development of atherosclerosis [37–40]. Accordingly, treatments for lowering lipids, autophagy enhancement and inflammation inhibition have been widely developed [24, 41, 42]. Currently, the most successful treatment are statins, which produce approximately a 30% reduction in the risk of cardiovascular events [43]. However, atherosclerosis is still the major cause of death and disability worldwide and is a great burden on society [44]. In clinical practice, drugs are usually applied to symptomatic patients. This fact forces us to confront the question of whether interventions against atherosclerotic factors at an earlier time point would alter the pathogenetic approach to atherosclerosis.

The present study revealed that inhibition of ox-LDL phagocytosis in the early stage of atherogenesis can significantly promote macrophage autophagic capacity, attenuate NLRP3 activity and cell apoptosis, and reduce foam cell formation. Although inhibition of ox-LDL phagocytosis in the advanced stage also enhanced macrophage autophagy compared with the control, autophagic capacity was attenuated and the number of foam cells was remarkably increased compared to the early stage. In addition, inhibition of ox-LDL phagocytosis in the advanced stage did not significantly change NLRP3 activation or cell apoptotic status compared with control cells, but their relative levels were much higher than in the early stage. These data suggest that lipid abnormality should be addressed in the early stage to maintain macrophage autophagic status and remove cellular lipid accumulation, thereby inhibiting inflammatory reactions, cell apoptosis and preventing macrophage foam cell formation. Otherwise, autophagy will largely have a damaging effect and fail to metabolize cellular lipids, leading to continuous and excessive accumulation of cellular lipids for a prolonged period. Subsequently, inflammatory reactions, development of foam cells and apoptosis would become irreversible even if treatments to lower lipids were administered. A growing body of research has shown that lipid metabolism disorders develop in early life, occurring in more than 70% of children and adolescents [45]. Consequently, some experts advocate early-life initiation of pharmacological interventions for dyslipidemia because early optimal lipid control leads to improved outcomes [46, 47]. These recommendations are consistent with the results of the current study. However, it still needs to be determined how patients most at risk should be identified and how early pharmacological intervention should be started.

Studies have demonstrated the crosstalk between inflammation, autophagy and apoptotic pathways [14, 48, 49]. This crosstalk plays an important role in atherogenesis. In the present study, application of si-NLRP3 in the early stage of atherogenesis enhanced autophagic capacity, reduced macrophage apoptosis and foam cell formation, and also significantly lowered NLRP3 expression. Although the use of si-NLRP3 in the advanced stage did not significantly change autophagy, apoptotic status or foam cell formation compared with the controls, the amount of ATG7, Beclin1, LC3 II/I and Bcl2 decreased, and the amount of *p*-mTOR, p62 and Bax obviously increased, compared to the early stage. This suggested that early intervention for inflammasome activation could not only help to alleviate inflammatory reaction efficiently but also enable recovery of autophagic status and dramatically reduce macrophage apoptosis and foam cell formation. In contrast, intervention against inflammasome activation in the advanced stage may aggravate autophagy dysfunction and poorly control apoptosis and foam cell formation because of unsuppressed inflammatory reactions. These results are in line with the literature [14, 48]. To some degree, the data also explain why the inflammatory signaling pathway is a promising target for alleviating atheropathogenesis [42, 50]. Nevertheless, similar problems need to be confronted with this approach: how to identify the initiation of atherosclerotic inflammation, the optimal start time for pharmacological therapy and how to coordinate lipid-lowering and anti-inflammatory treatments.

### Study strengths and limitations

The strengths of this article are: (1) This is first time that a comprehensive comparison study has been conducted evaluating macrophage autophagy, inflammatory response and apoptotic status after interventions to stop lipid damage in the early and advanced stages of atherogenesis; (2) this study is the first to show that the timing of interventions against inflammasome activation may control the intensity of inflammation, autophagy and apoptosis in macrophages.

There are also some limitations of this study. The study was carried out via in vitro experiment, and clinical treatment by pharmacological intervention was not observed. It is much easier to manage intervention for a specified time in a cell model while the initiation and progression of atherosclerotic pathogenesis is more complicated and difficult to detect in an animal model or clinical patient. Nonetheless, to the extent possible, further investigation should be conducted in an animal model, and additional clinical data should be collected to verify the timing of pharmacological treatments for lipid abnormalities and the ensuing atherosclerotic inflammation.

## Conclusions

**Fig. 7:**
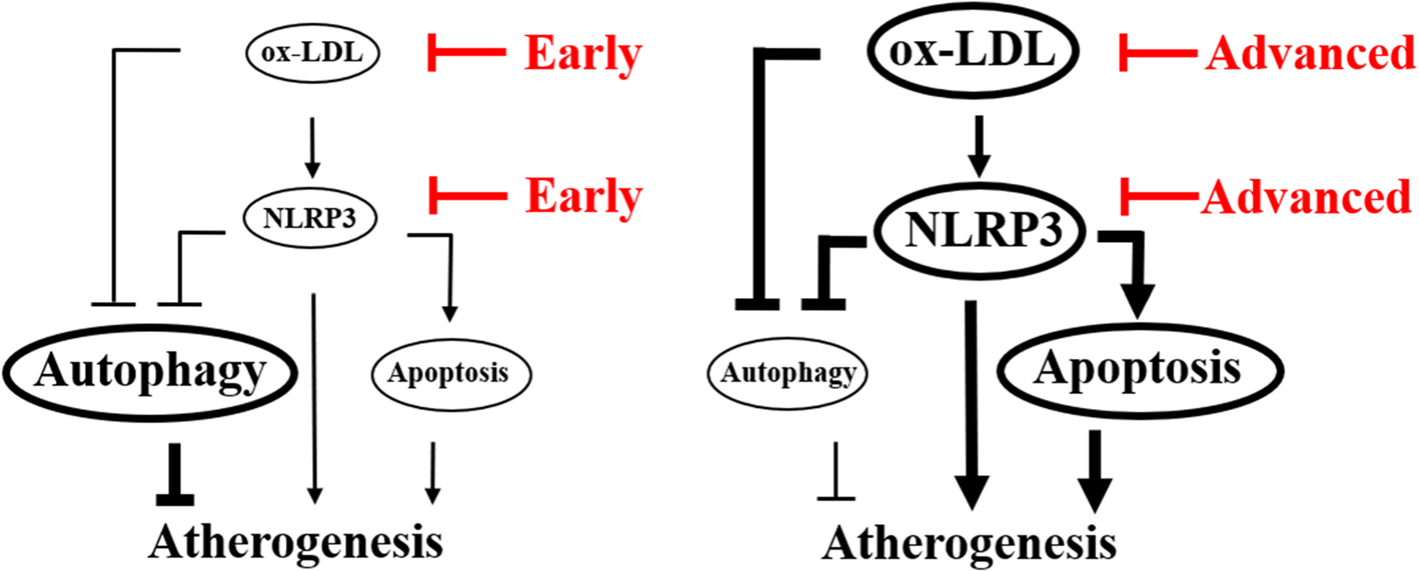
Schematic diagram

The current work has revealed that treatments for lipid disorders at an early stage may help maintain macrophage autophagic status, inhibit inflammatory reactions, and reduce macrophage apoptosis and foam cell formation. Similarly, interventions against inflammasome activation in the early stage may efficiently alleviate inflammatory reactions, improve autophagic status and simultaneously suppress macrophage apoptosis and foam cell formation. The data suggest that lipid abnormalities and atherosclerotic inflammation in patients should be detected as early as possible to ensure that drugs can be administered in time to improve patient prognoses (Fig. 7) [51]. Due to the subtle issues in identifying atherosclerotic factors and the optimal start time for pharmacological therapy in clinical practice, a healthy lifestyle should always be adopted for the primary prevention of atherosclerosis.

## Supplementary Information

**Additional file 1. FigS1. **Intracellular lipid accumulation is alteredafter exposure to different concentrations and durations of ox‐LDL incubation.

## Data Availability

All data generated or analyzed during this study are included in this published article.

## Abbreviations

ox-LDL: Oxidized low-density lipoprotein
TEM: Transmission electron microscope
NLRP3: Nucleotide-binding oligomerization domain-like receptor containing pyrin domain 3
ORO staining: Oil Red O staining
PMA: Phorbol-12-myristate-13-acetate
TUNEL: Terminal deoxynucleotidyl transferase dUTP nick-end labeling
PBS: Phosphate buffer saline
