# Supplementary material for: Intervention time decides the status of autophagy, NLRP3 activity and apoptosis in macrophages induced by ox‐LDL
Source: Lipids Health Dis. 2022 Oct 25;21:107. doi: 10.1186/s12944-022-01714-x (PMC9594915; doi:10.1186/s12944-022-01714-x)
Supplement: Supplementary file 1 — Additional file 1. FigS1. Intracellular lipid accumulation is alteredafter exposure to different concentrations and durations of ox‐LDL incubation. [file 12944_2022_1714_MOESM1_ESM.pdf]

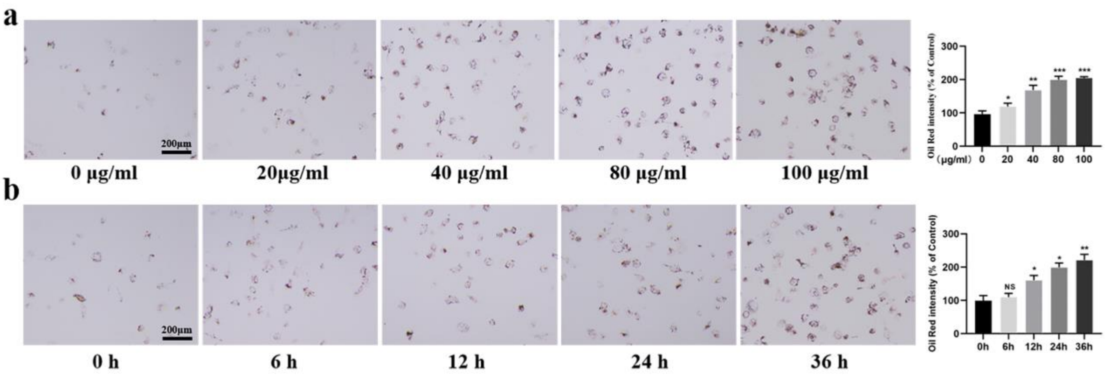

Fig S1 Intracellular lipid accumulation is altered after exposure to different concentrations and durations of ox-LDL incubation. **a** Macrophages were stimulated with different doses of ox-LDL (0, 20, 40, 80 and 100 μg/ml) for 24 h, and intracellular lipid droplets were visualized by ORO staining. **b** Intracellular lipid droplets were visualized by ORO staining after the cells were treated with 80 μg/ml ox-LDL for different durations (0, 6, 12, 24 and 36 h). Scale bar = 200 μm. Data represent means ± SEM from triplicate independent experiments. *P < 0.05; **P < 0.01; ***P < 0.001.
